# 3D anatomy of the heart in teaching: differentiating physiological and pathological changes in wild ungulates in central Europe

**DOI:** 10.3389/fvets.2026.1811948

**Published:** 2026-05-11

**Authors:** Klára Matějka Košinová, Alena Říhová, Rostislav Linda, Jan Cukor

**Affiliations:** 1 Department of Game Management and Wildlife Biology, Faculty of Forestry and Wood Sciences, Czech University of Life Sciences Prague, Prague, Czechia; 2 Forestry & Game Management Research Institute, Jíloviště, Czechia

**Keywords:** 3D model, cardiac anatomy, CT scanner, Education, *Os cordis*

## Abstract

**Introduction:**

Accurate knowledge of the heart's anatomy and its physiological variability in wild ungulates is essential for the correct interpretation of necropsy and imaging findings, veterinary diagnostics, and teaching in fields focused on hunting and wildlife management. One of the most frequently misinterpreted structures is the *Os cordis*, whose occurrence, morphology, and clinical significance in most species of wild ungulates have not yet been sufficiently studied by modern imaging methods. Therefore, the main aim of this study was to systematically describe the occurrence, localization, and morphometric characteristics of the *Os cordis* of the most common Central European wild ungulates using computed tomography (CT) and to verify the contribution of CT-based 3D models for teaching and distinguishing between physiological and pathological changes in the heart.

**Methods:**

A total of 131 hearts of roe deer (*Capreolus capreolus*), red deer (*Cervus elaphus*), fallow deer (*Dama dama*), mouflon (*Ovis gmelini musimon*), and wild boar (*Sus scrofa*) were examined using the multidetector CT.

**Results:**

The presence, location, density, volume, and size of the *Os cordis* were evaluated. Digital 3D models were created from DICOM data and used for both morphological analysis and teaching purposes. *Os cordis* was found in all ruminants studied, *Os cordi*s was found in 43 out of 45 roe deer, in 32 out of 36 mouflons, in 17 out of 18 red deer, and in all fallow deer hearts, while it was not found in wild boar. The density and size of the *Os cordis* increased significantly with age and differed between sexes and species, while volume was primarily influenced by body weight. The integration of 3D models into teaching led to a significant improvement in students' ability to correctly locate and identify the *Os cordis*.

**Discussion:**

The results demonstrate that CT and 3D modeling are effective tools for cardiac morphology research, veterinary diagnostics, and modern teaching.

## 1 Introduction

The development of teaching methods across disciplines is a desirable trend, although it is still underutilized in some contexts. It is prudent that teaching methods correspond to the times and are able to educate more effectively and highlight important findings, not only for the purpose of teaching veterinary medicine, but also for other disciplines addressing, for example, wild animals. Wildlife management and conservation stresses the education on a multidisciplinary scale, including ecology and landscape management. The focus is on improving habitats for wildlife and understanding relationships among different parts of ecosystems, with an emphasis on wildlife, particularly game species (1–4). At the same time, there is a growing call to regulate the increasing number of wild ungulates in many regions, especially in Central Europe and other countries worldwide (5–8). A population growth and local overabundance is not limited only to native wild ungulate species, such as European red deer (*Cervus elaphus*), wild boar (*Sus scrofa*), or roe deer (*Capreolus capreolus*), but also to introduced ones. In Central Europe, they include fallow deer (*Dama dama*) and mouflon (*Ovis gmelini musimon*) (9). However, management strategies should be based on biological knowledge of particular wildlife species. Awareness of anatomy and recognition of potential wildlife diseases is a fundamental milestone in creating effective and sustainable wildlife management. This requires the ability to distinguish between physiological anatomical variations and actual pathological changes- a neglected skill that is often limited in wild species due to a lack of systematically collected (10), high-resolution anatomical data.

With the advancement of modern technologies, the acquisition of knowledge is evolving and offers new, previously unimaginable opportunities for education. One method is CT scanning, which provides high-resolution anatomical data that can then be converted into 3D digital models (e.g., STL) and printed if necessary (11). CT scanning offers a unique, non-invasive method for visualizing mineralized structures and soft tissues directly in their natural environment (12), thereby serving as a crucial bridge between anatomical research and its accurate interpretation in both physiological and pathological contexts (13). For veterinary students, 3D printed models based on CT scans have been shown to improve the ability to identify vertebral fractures and understand the anatomy of the spine (14). 3D models derived from CT data are also used to teach the complex anatomy of the heart, skull, bones, and other structures (15). These 3D models are created using well-characterized datasets, they allow students to repeatedly examine anatomically accurate representations, which facilitates the recognition of subtle morphological differences and enhances their ability to distinguish physiological structures from pathological findings (13). For teaching and data sharing purposes, it is essential to convert DICOM data from CT scans into manipulable 3D models (segmentation, retopology, and export for 3D printing). Recent studies demonstrate a standardized workflow: beginning with CT scanning, followed by software-based segmentation (thresholding and manual refinement), and concluding with volume/density analysis and the 3D printing of physical models (16). The integration of anatomical data obtained from imaging studies into educational tools therefore represents not only a technological advancement but also a necessary step toward evidence-based education that takes into account real biological variability and improves diagnostic reasoning (17, 18).

In wildlife management (19) and especially in veterinary disciplines, the anatomical study of the cardiac skeleton and associated structures (e.g., *ossa cordis* and *cartilago cordis*) in wild ungulates is essential for understanding the functional morphology of the heart, interpreting pathological findings, and assessing the health status of individuals and populations in the wild (20). The cardiac skeleton is composed of fibrocartilaginous and hyaline tissues, providing mechanical support to the cardiac valvular apparatus, atrioventricular rings, and conductive tissue, while in some taxa, heterotopic mineral formations known as *ossa cordis* and *cartilago cordis* may develop within it (21). This complex of anatomical structures influences the biomechanics of cardiac contraction, which maintains the heart's shape during systole (22), and may be related to physiological and pathological processes in the hearts of mammals.

The anatomy and pathology of the cardiac skeleton are well described in some farm animals, such as sheep and goats (23–25), cows (13, 26), and horses (27). This not only extends to camels (28), elephants (29), giraffes (30), or water buffaloes (31), but also carnivores, such as otters (32) or dogs and cats (33, 34). Contrastingly, in many non-ruminant mammals, including humans (35) and some primates (36), *Os cordis* is either rare or its occurrence is primarily associated with pathological processes, such as atherosclerosis, myocardial fibrosis, or dystrophic ossification. Furthermore, for most species of wild ungulates, there is a lack of systematic data supported by imaging methods that would allow normal (physiological) variations to be distinguished from actual pathological changes. This significantly limits the ability of wildlife managers, veterinarians, and students to reliably interpret findings from necropsies or imaging methods. This ambiguity is further reflected in the classroom, where students are often unable to develop a spatial and functional understanding of these structures and, as a result, have difficulty distinguishing normal anatomical variability from pathological changes (17).

The aim of this study is therefore: (i) to systematically describe the occurrence, localization, and morphometry of the *Os cordis* in the most common species of ungulates in Central Europe—roe deer (*Capreolus capreolus*), red deer (*Cervus elaphus*), fallow deer (*Dama dama*), mouflon (*Ovis gmelini musimon*), and wild boar (*Sus scrofa*) using a CT scanner; (ii) to analyze the relationships between the morphology of the *Os cordis* and the biological parameters of individuals (weight, age, sex); (iii) to create and validate digital 3D models of hearts as teaching aids and illustrate their contribution to distinguishing between physiological and pathological changes.

## 2 Materials and methods

### 2.1 Heart preparation

For the research portion, 45 hearts from roe deer (*Capreolus capreolus*), 36 from mouflon (*Ovis gmelini musimon*), 13 from fallow deer (*Dama dama*), 18 from red deer (*Cervus elaphus*), and 19 hearts from wild boar (*Sus scrofa*) were used. In all cases, the hearts were obtained from game hunted in accordance with standard game management practices, which are based on Hunting Act No. 449/2001 (37) and related implementing regulations.

The hearts were removed from the carcasses and separated so as not to damage the heart tissue. The hearts were then soaked in water to ensure the most effective bleeding possible. Ethanol was used as a fixative medium for scanning the hearts to detect the presence of *Os cordis* or pathological changes. The use of water was ruled out due to its tendency to release residual blood from the tissue, which makes it difficult to determine density interfaces. Ethanol fixes the tissue effectively and helps to improve the quality and contrast of the output image. The use of alcohol achieved a greater contrast between the myocardial tissue and the fluid in the hollow organs.

### 2.2 CT scanning

A Siemens Somatom Scope VC40 MDCT scanner was used for the procedure. The hearts were placed in plastic cups with the *apex cordis* pointing downwards (caudally) toward the bottom. The anterior wall of the heart was placed in the direction outside the gantry (localization using the *truncus pulmonaris*).

Range of density values:

- Alcohol: −150 to −200 HU (Hounsfield units)
- Tissue: 20–50 HU

**Figure 1:**
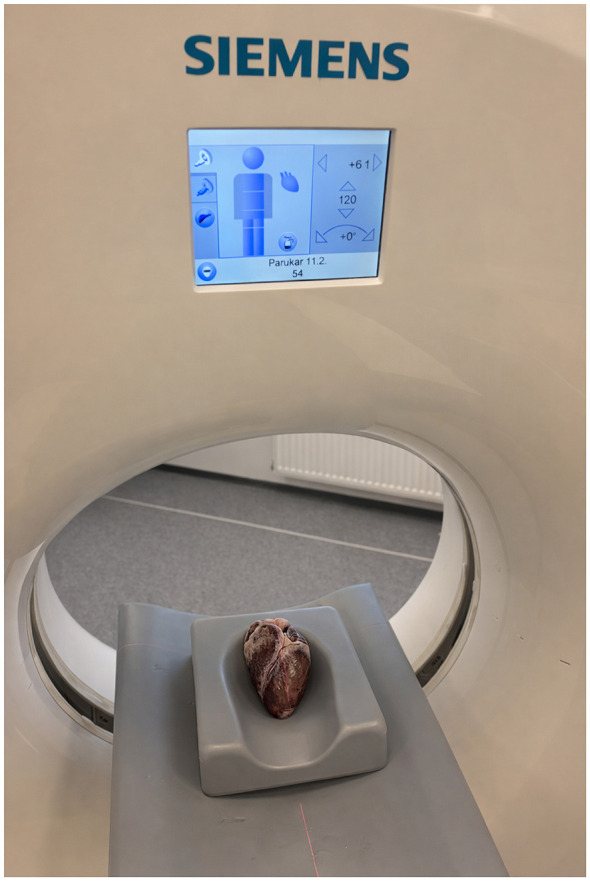
The process of wild boar heart CT scanning–positioning of the heart in the gantry during imaging.

In order to create 3D models, the hearts were placed in ethanol for 12 h, then removed and scanned on a pad (Figure 1). Although the cup ensures a stable position, it interferes with the overall appearance of the scanned 3D object. Separating the object from the cup was also problematic.

#### 2.2.1 Acquisition parameters

Samples were scanned using parameters that were consistent for all samples regardless of type (38–40) (Table 1).

**Table 1:** Scanning parameters.

*Parameter*	*Value*
*X–ray tube current*	82 mAs
*X–ray tube voltage*	110 kV
*scanning time*	13.1 s with a 3–s delay
*slice*	0.6 mm
*collimation*	16 × 0.6 mm
*X–ray tube rotation*	1.0 s/rotation
*pitch factor*	0.8

Image (reconstruction) parameter settings and postprocessing.

The first reconstruction corresponds to the acquisition parameters (Table 2).

**Table 2:** The first reconstruction parameters.

*Parameter*	*Value*
*layer width*	1
*Layer overlap*	0.6
*First reconstruction kernel*	D45s medium sharp
*slices*	2 mm with a 1.4 overlap
*window*	Mediastinum
*field of view*	according to the size of the object of interest

The second reconstruction was for determining the location and measuring the size of the *Os cordis* (Table 3).

**Table 3:** The second reconstruction parameters.

*Parameter*	*Value*
*Layer overlap*	0.6
*slices*	1 mm

Values were adjusted for 3D MPR to determine the standard measurement plane of the heart. The measurement plane of the heart was determined—Apicobasal axis, comparison on the sagittal slice, orientation of the interventricular septum, and axial slice set by a perpendicular transverse slice to the interventricular septum.

#### 2.2.2 *Os cordis* detection and measurement

**Figure 2:**
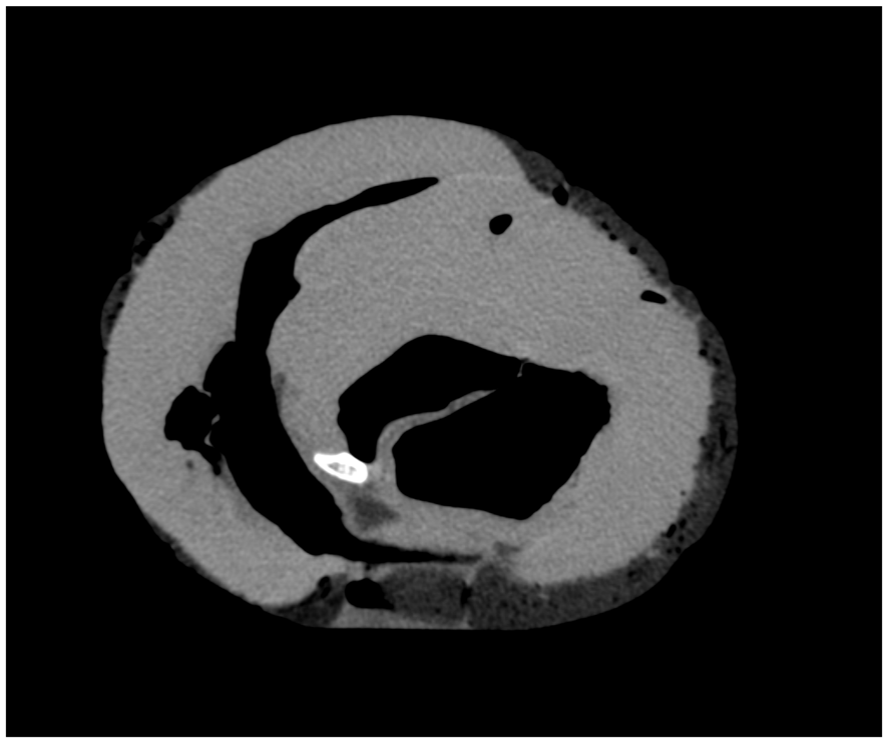
*Os cordis* view in cardiac protocol.

**Figure 3:**
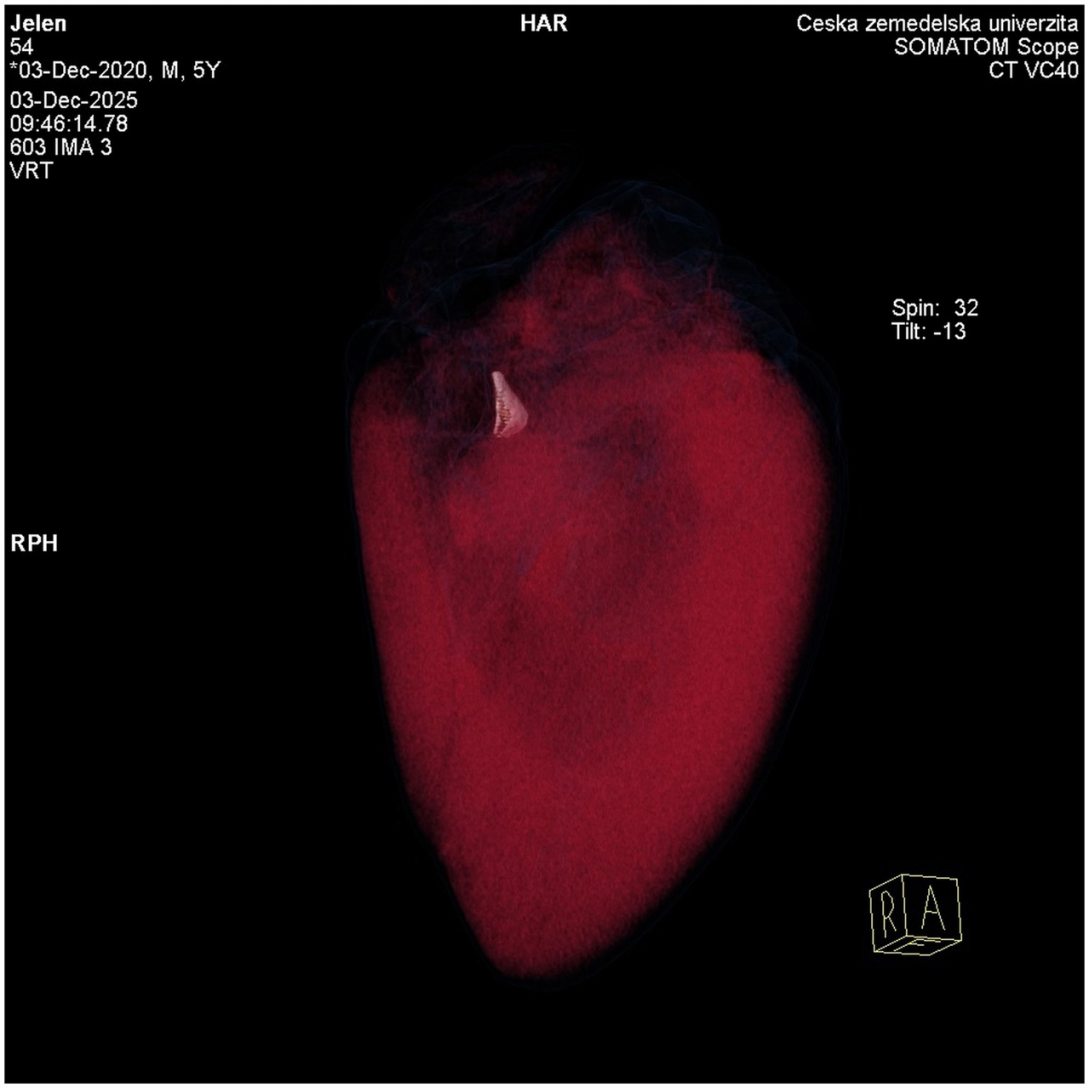
VRT free view of the red deer heart with *Os cordis*. The Volume Rendering Technique, i.e., the method of three-dimensional reconstruction, was corrected and performed by adjusting the window settings. With this setting, the outline of the heart surface is clearly visible and the *Os cordis* of the heart stands out distinctly, with a significantly higher density than the surrounding myocardium.

The bone display protocol was selected for measuring *Os cordis*. MPR Thicknes was selected for localization to enable verification according to the cardiac protocol (Figure 2), and, in some cases, a VRT free view (Figure 3).

The source data from the first reconstruction was used for CT volumetry (41). The measurement was performed automatically using the upper and lower density thresholds of the target object. When measuring the total volume of the *Os cordis*, its average density was measured in the same way. According to data obtained from the 3D Slicer (42), the voxel size in the protocol used is 0.248 × 0.248 × 0.600 mm, which corresponds to a high-resolution anisotropic resolution in the slice plane.

Threshold settings:

−50 HU to 500 HU for the total volume of the heart, including the *Os cordis*
- 100 HU to 500 HU for measuring the volume of the Os cordis

Specific settings for individual density thresholds by species–*Dama dama*: 114.05–449 HU, *Ovis gmelini musimon*: 111.13–466.64 HU, *Capreolus capreolus*: 119.35–329 HU, *Cervus elaphus*: 115.67–969.86 (density thresholds were manually adjusted in this case).

To verify the accuracy of the measurements, the density values of the reference materials—air and water—measured on a CT calibration phantom were checked. The measured values corresponded to the reference values for those materials (43). All volume and density measurements were performed by two independent observers.

### 2.3 Correction and preparation of the 3D model

In the initial phase, the scanned 3D object was exported in DICOM format (44). The object was then imported into 3D Slicer (42), which was used to export it to STL format. Using Zeiss Inspect Optical 3D 2026 (45), basic editing of 3D models in STL format was performed. Initially, the image was cleaned of any artifacts captured by the model–no artifacts were observed at the interface between ethanol and tissue. Any artifacts that did occur were, in some cases, caused by the CT scanner table. Subsequently, the mesh was polygonized to maximum detail, and the data was then exported. The 3D model prepared in this way was further edited in the online version of the Sketchfab 2025 program and platform. In the Edit 3D Settings section, the final adjustments were made to the object, specifically focusing on the model's focus, texturing, and coloring. The models were later published and made publicly available on this platform.

To create an illustration of the basic anatomical features of the heart for determining the position of the right and left fibrous triangles (trigonum fibrosum dextrum et sinistrum) and the axis of the heart, a 3D model was modified using Blender 5.0 (46). To achieve authenticity, a texture was created and applied using photographs of the same real heart in the same position.

### 2.4 The use of 3D models in lessons

As part of the Hunting course, 3D models were implemented to train third-year students in the Game Management and Wildlife Environment Bachelor's program. The curriculum focuses on essential skills such as meat hygiene, early disease recognition, and anatomical preparation, including the identification of minor *Os cordis*. A total of 41 students (20 women and 21 men) participated in the first integration of 3D models for teaching this field. In the first lesson, students were introduced to the principles of CT scanner operation and the basic application possibilities in the field.

During the 90-min session, students were introduced to the provided 3D models and received a briefing on the unique anatomical features of various ungulate hearts. Following these instructions, they spent 30 min in independent study, utilizing the models to deepen their understanding.

At the end of the lesson, students took a test consisting of four separate parts, which were evaluated. In the first section, they were instructed to describe the anatomy of the heart through photographs. Each participant had to determine the orientation of the heart in a space relative to the pulmonary trunk. To pass this part, examinees had to specify the right and left ventricles, the apex of the heart, the right and left atria, the aorta, and the pulmonary trunk. In an area oriented toward the pulmonary trunk, students had to further specify both ventricles, the interventricular septum, the mitral valve, and then the *trigonum fibrosum dextrum et sinistrum* area. In the second step, the description of the location of the *Os cordis*–*anuli fibrosi*, and then the *trigonum fibrosum dextrum et sinistrum* was required. The third part tested the heart of *Capreolus capreolus*, where the students determined the presence and location of the *Os cordis* by palpation. In the fourth part of the test, participants performed a dissection to isolate the *Os cordis* from the surrounding the muscle tissue, ensuring the bone remained entirely undamaged.

The results were then compared with those from the previous year that underwent the same test. Preparation for the earlier test took place in the same time frame, but without the use of 3D models, only through a standard PowerPoint presentation, using a simple visual of the heart with a description. The previous year's class also had a total of 41 students, 15 women and 26 men. The test format was identical for both years.

### 2.5 Statistical analysis

For the assessment of the effects of species, sex, age, and weight on *Os cordis* density, a volume and size, a linear model was used. For size, the product of width and height was used (size is in cm^2^), but this metric does not describe the actual area of *Os cordis*, but rather a projected bounding–dimension metric.

The final model was constructed using backward selection in all cases. Backward selection included the assessment of the VIF scores, and predictors with a VIF > 4 were excluded from the model. For each model estimate, 95% CI is reported altogether with Cohen's f^2^ metric of local effect size.

Multiple comparisons of the particular metric between species were conducted using estimated marginal means and the Tukey method. Results are visualized using a bar plot with error bars and indices showing the results of *post-hoc* multiple comparisons (bars sharing the same index are not significantly different, whereas bars with different indices differ significantly).

The comparison of student examination success rates was performed using the Fisher exact test. The results are depicted using a grouped bar plot.

All statistical computations were performed in R software (47) using an alpha level of 0.05. Marginal means and their comparison were computed using the package “emmeans” (48) and “multcomp” (49). VIF score was computed using the “performance” package (50). Plots were created using the “ggplot” (51) package. Cohen's f^2^ was computed using “effectsize” package (52).

## 3 Results

### 3.1 Os cordis

#### 3.1.1 Occurrence of *Os cordis*

*Os cordis* was recorded in all polygastric species, i.e., *Cervus elaphus, Capreolus capreolus, Dama dama*, and *Ovis gmelini musimon*. In this study, *Os cordis* was detected in 17 of 18 samples (94%) for *Cervus elaphus*, 43 of 45 samples (96%) for *Capreolus capreolus*, all 13 samples (100%) for *Dama dama*, and 32 of 36 samples (89%) for *Ovis gmelini musimon*. In contrast, not a single case was recorded in *Sus scrofa*. *Ovis gmelini musimon* showed the lowest incidence rate among polygastric species, with four individuals without *Os cordis* being in the age range of 2–8 months and one individual being 60 months old. In *Capreolus capreolus*, the *Os cordis* was not found in a total of two animals aged 12 and 24 months. In *Cervus elaphus*, the *Os cordis* was found to be missing in a 12-month-old specimen. In *Dama dama, Os cordis* was present in all individuals.

#### 3.1.2 Localizing the *Os cordis*

**Figure 4:**
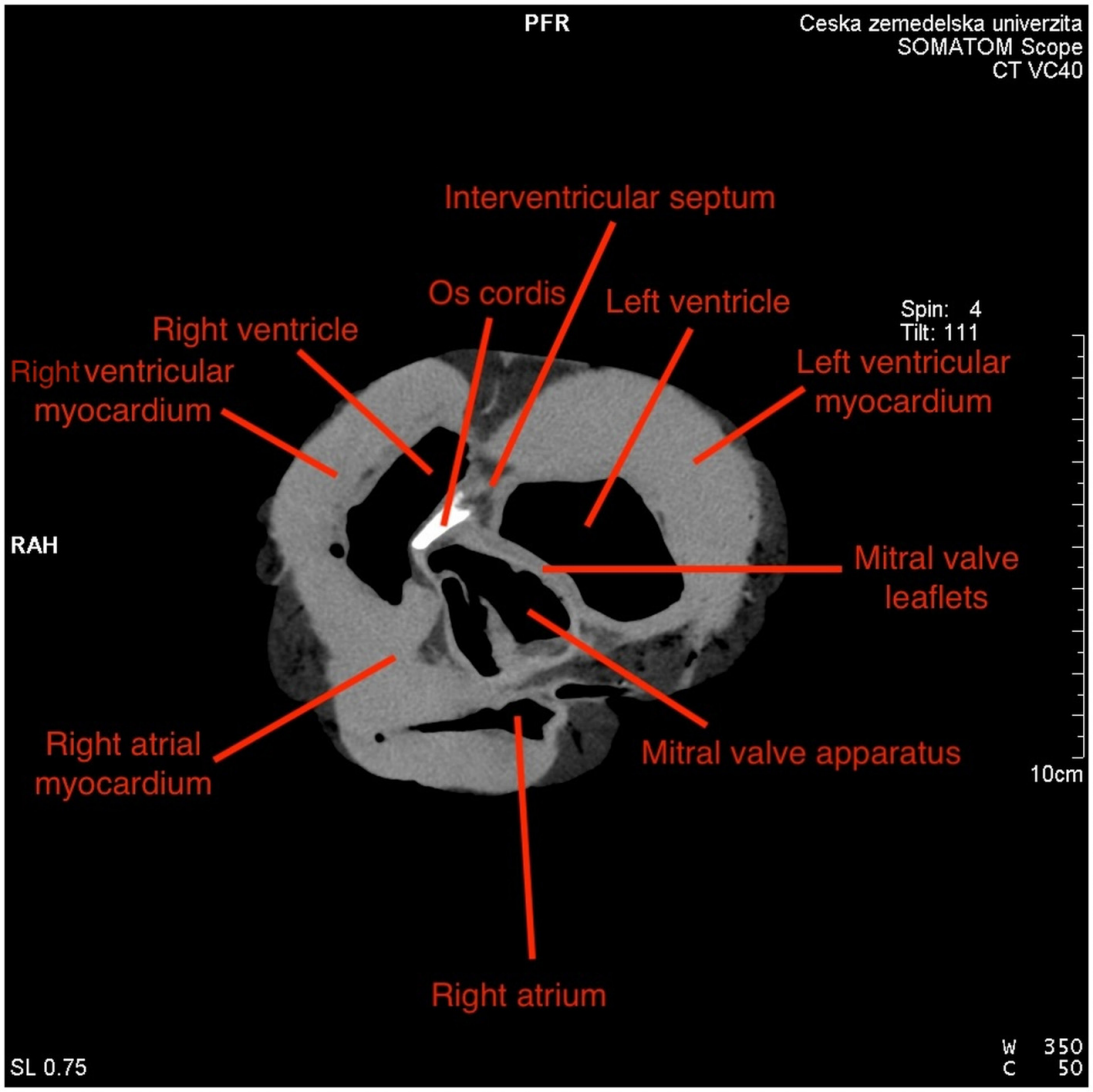
*Os cordis* localization in *trigonum fibrosum dextrum* of *Cervus elaphus* with anatomical description.

*Os cordis* was observed exclusively in the *trigonum fibrosum dextrum* (Figure 4) of selected species, even in older animals. No ossification occurred in the *trigonum fibrosum sinistrum*.

#### 3.1.3 Dependence of *Os cordis* density on species, sex, age, and weight

The dependence of density of *Os cordis* on species, sex, age, and weight was analyzed by a linear model. Firstly, a full model containing all mentioned descriptors was created. In this case, the effect of weight was not statistically significant and was excluded from the final model. The results of final model are presented in Table 4.

**Table 4:** The results of the linear model of *Os cordis* density based on species, sex, and age.

*Predictors*	Response—*Os cordis* density (HU)					
	*Estimate*	*SE*	*95% CI*	*f ^2^*	*t*	*P*
(Intercept)	136.44	8.13	120.3–152.6		16.79	**<0.001**
Species [*Cervus elaphus*]	36.38	9.45	17.6–55.1	0.32	3.85	**<0.001**
Species [*Dama dama*]	61.90	10.18	41.7–82.1		6.08	**<0.001**
Species [*Ovis gmelini musimon*]	24.53	7.85	9.0–40.1		3.12	**0.002**
Sex [female]	−17.55	6.79	−31.0– −4.1	0.07	−2.58	**0.011**
Age (months)	0.94	0.16	0.6–1.3	0.33	5.74	**<0.001**
Observations	105
R^2^	0.42

Bold font indicates statistically significant differences in values.

The effect of sex was statistically significant, where the model density value for males was 17.55 HU greater than that of females. The effect of age was also highly significant, where on average, *Os cordis* density increased by 0.94 HU with each month of the individual's age.

**Figure 5:**
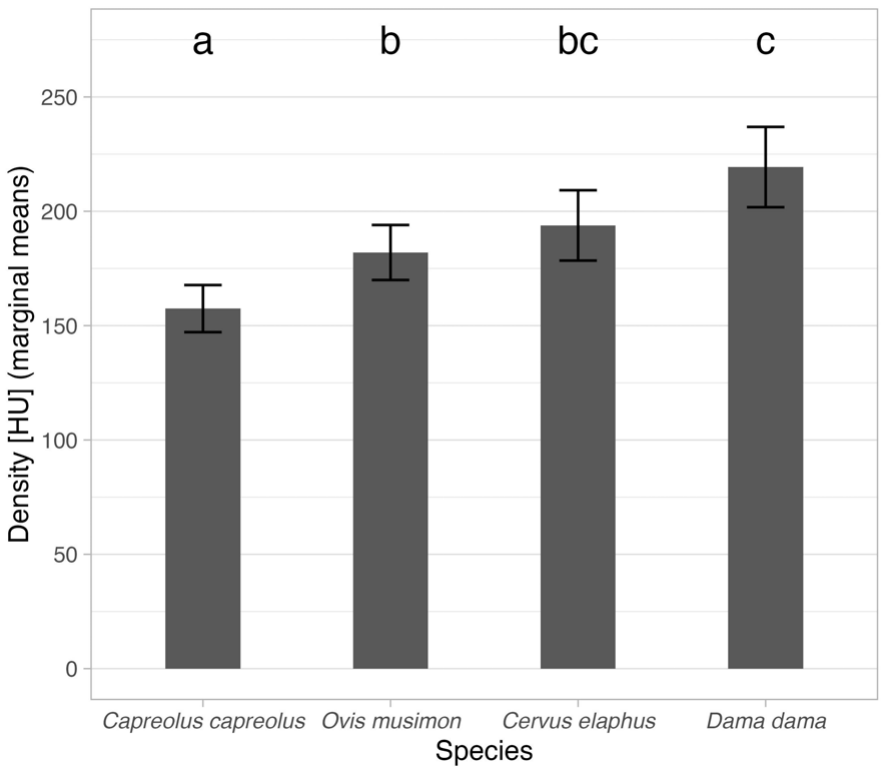
Estimated marginal mean (derived from the model in Table 4) values for analyzed species. Error bars depict 95% CI. Indices above each bar depict statistical homogeneity (variants with different indices have significantly different values and vice versa).

Further multiple comparisons of species (using marginal mean values) showed significant differences between *Os cordis* density for *Capreolus capreolus* (marginal mean = 157 HU, 95% CI 147–168 HU) from all others and also between the values for *Ovis gmelini musimon* (182 HU, 95% CI 170–194 HU) and *Dama dama* (219 HU, 95% CI 202–237 HU). Marginal means for analyzed species with 95% CI and multiple comparisons results are graphically depicted in Figure 5.

#### 3.1.4 Dependence of *Os cordis* volume on species, sex, age, and weight

Also, the dependence of volume (in cm^3^) of *Os cordis* on individual species, sex, age and weight was analyzed by a linear model. At first, a full model containing all mentioned descriptors was created as in previous case. For modeling, only species and weight showed to have significant effects on *Os cordis* volume. The results of final model are presented in Table 5.

**Table 5:** The results of the linear model of *Os cordis* volume based on individual species and weight.

*Predictors*	Response—*os cordis* volume (cm^3^)					
	*Estimate*	*SE*	*95% CI*	*f ^2^*	*t*	*P*
(Intercept)	0.00	0.02	−0.05–0.05		−0.15	0.881
Species [*Cervus elaphus*]	−0.11	0.05	−0.22–0.00	0.51	−2.06	**0.042**
Species [*Dama dama*]	0.08	0.05	−0.02–0.17		1.52	0.131
Species [*Ovis gmelini musimon*]	0.04	0.03	−0.01–0.10		1.52	0.133
Weight (kg)	0.01	0.01	0.00–0.01	0.29	5.38	**<0.001**
Observations	104
R^2^	0.446

Bold font indicates statistically significant differences in values.

Individual weight showed a highly significant result (*P* < 0.001); on average, the volume of *Os cordis* increased by 0.1 cm^3^ with each kg of body weight.

**Figure 6:**
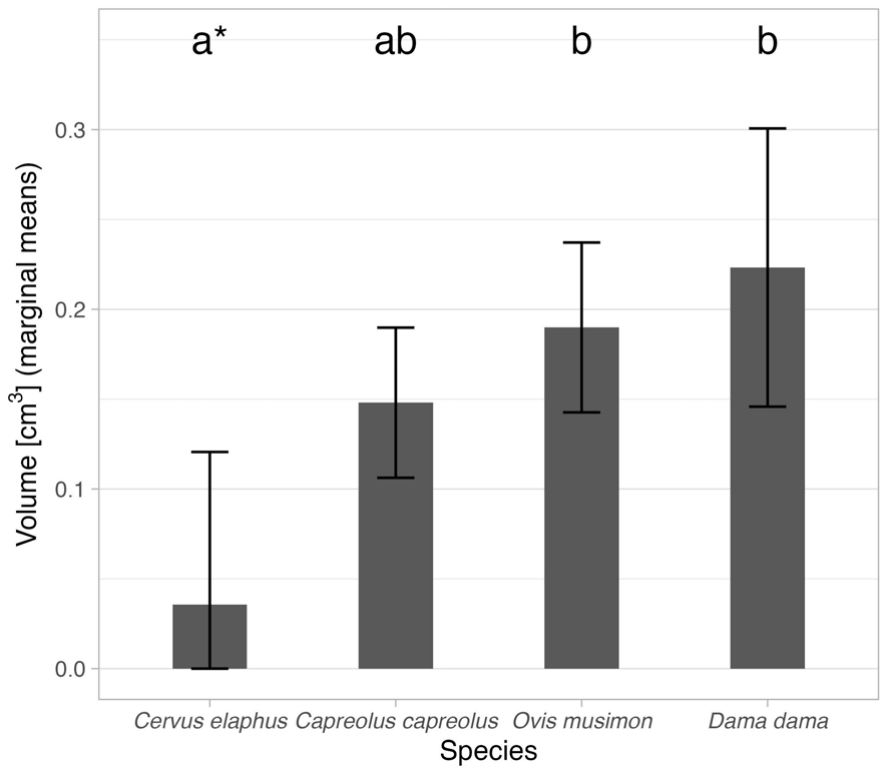
Estimated marginal mean (derived from the model in Table 5) values of *Os cordis* volume for the analyzed species. Error bars depict 95% CI. Indices above each bar depict statistical homogeneity (variants with different indices have significantly different values and vice versa). In the case of *Cervus elaphus*, the lower limit of the error bar was truncated at zero, as volume cannot take negative values. The affected variant is marked by “^*^” in the plot to emphasize this adjustment.

Multiple comparisons of species (using marginal mean values) showed significant differences between *Os cordis* volume for *Cervus elaphus* (marginal mean = 0.04 cm^3^, 95% CI 0–0.12 cm^3^; the actual lower CI limit was below zero—confidence intervals extending below zero were truncated at zero as volume cannot take negative values), *Ovis gmelini musimon* (marginal mean = 0.19 cm^3^, 95% CI 0.14–0.24 cm^3^), and *Dama dama* (marginal mean = 0.22 cm^3^, 95% CI 0.15–0.30 cm^3^). Marginal means for analyzed species with 95% CI and multiple comparisons results are graphically depicted in Figure 6.

#### 3.1.5 Dependence of size on species, sex, age, and weight

For *Os cordis* size, which was computed as its projected width × height (in cm), a linear model was constructed to assess its dependence on individual species, sex, age, and weight. At first, a full model containing all the mentioned descriptors was created. All predictors showed significant effects on *Os cordis* size, but weight was excluded from the model because of a relatively high correlation (VIF = 5.85) with other factors. The results of the final model are presented in Table 6.

**Table 6:** The results of the linear model of the *Os cordis* size based on individual species sex, age, and weight.

*Predictors*	Response—*os cordis* size (w × h in cm)					
	*Estimates*	*SE*	*95% CI*	*f ^2^*	*t*	*P*
(Intercept)	0.16	0.10	−0.05–0.36		1.54	0.127
Species [*Cervus elaphus*]	0.45	0.12	0.21–0.69	0.68	3.78	**<0.001**
Species [*Dama dama*]	1.07	0.13	0.82–1.33		8.33	**<0.001**
Species [*Ovis gmelini musimon*]	0.20	0.10	0.00–0.39		1.96	0.052
Sex [female]	−0.24	0.09	−0.41– −0.06	0.08	−2.74	**0.007**
Age (months)	0.01	0.01	0.01–0.02	0.43	6.55	**<0.001**
Observations	105					
R^2^	0.543					

Bold font indicates statistically significant differences in values.

On average, the size (projected – width × height, see Material and Methods) of *Os cordis* was 0.24 cm^2^ smaller in case of females compared to males. With each month of age, *Os cordis* size increased by 0.01 cm^2^.

**Figure 7:**
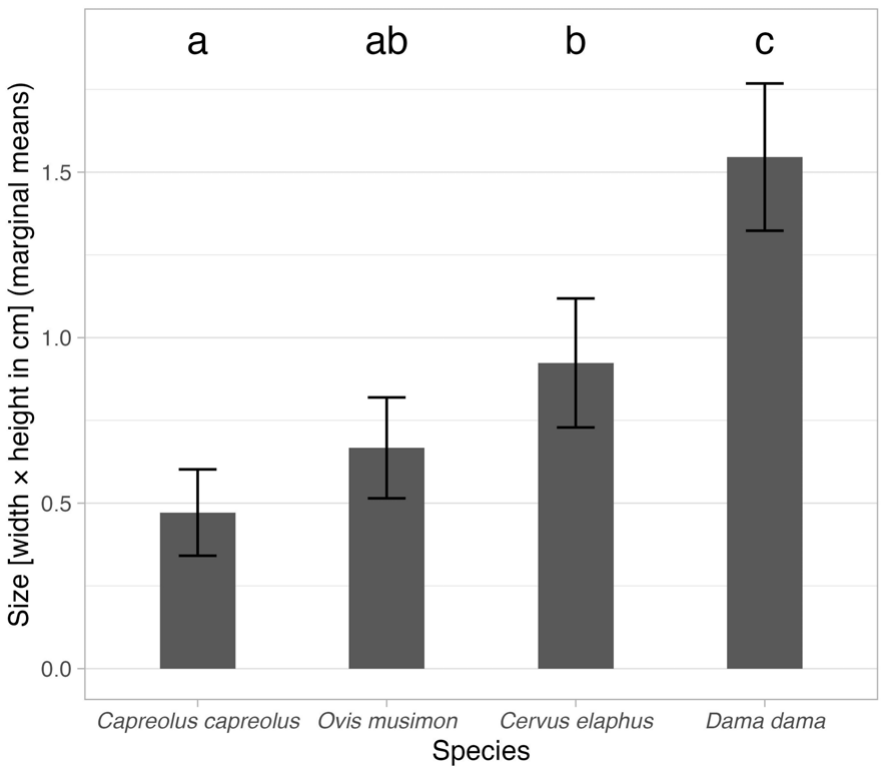
Estimated marginal mean (derived from the model in Table 6) values of *Os cordis* size for the analyzed species. Error bars depict 95% CI. Indices above each bar depict statistical homogeneity (variants with different indices have significantly different values and vice versa).

Multiple comparisons of species (using marginal mean values) showed significant differences between *Os cordis* size for *Dama dama* (marginal mean = 1.55 cm^2^, 95% CI 1.32–1.77 cm^2^) and all other species. The size of *Os cordis* was also statistically different between *Capreolus capreolus* (marginal mean = 0.47 cm^2^, 95% CI 0.34–0.60 cm^2^) and *Cervus elaphus* (marginal mean = 0.92 cm^2^, 95% CI 0.73–1.12 cm^2^). Marginal means for the analyzed species with 95% CI and multiple comparisons results are graphically depicted in Figure 7.

### 3.2 Applications for teaching

**Figure 8:**
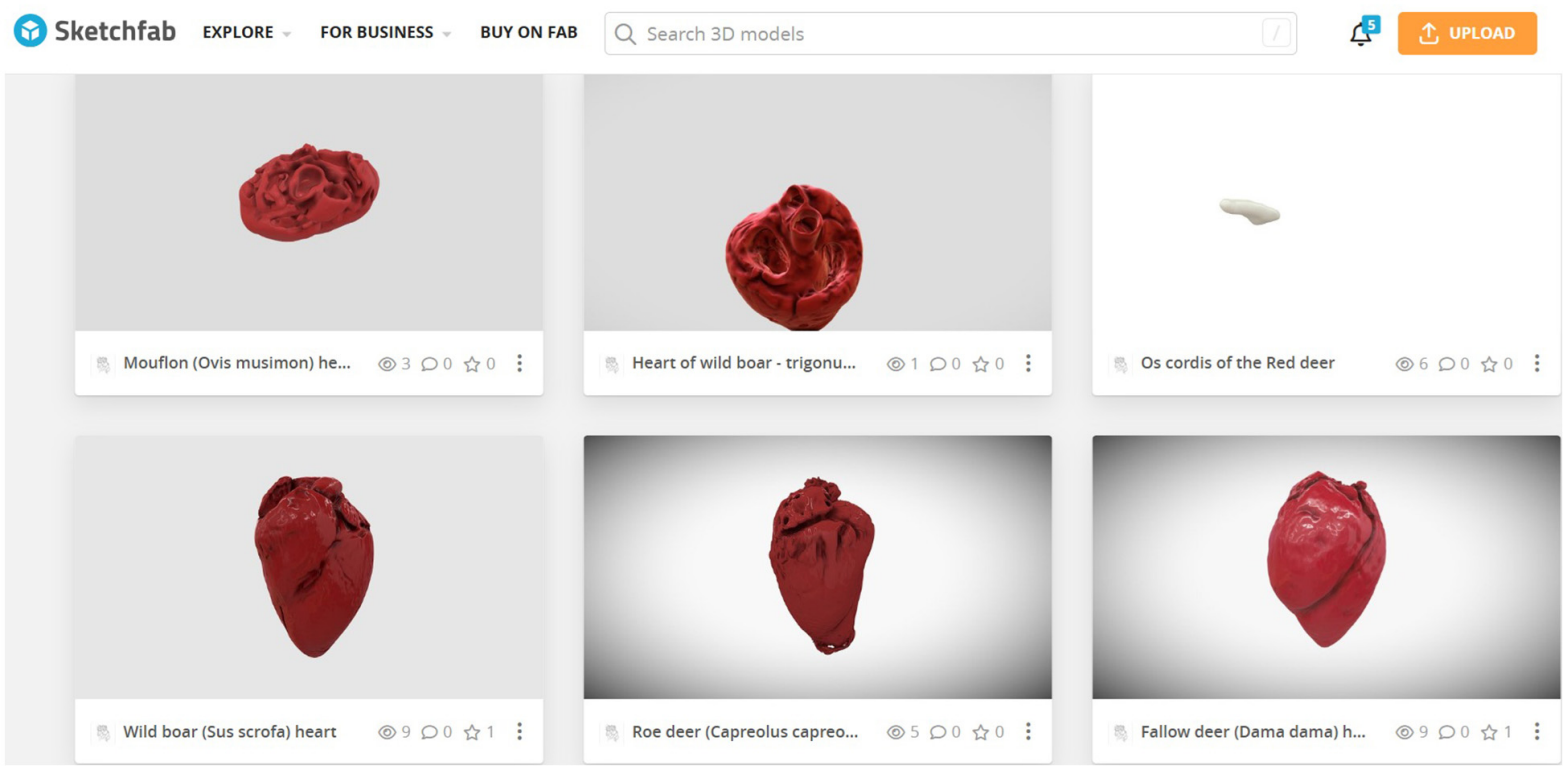
Skecthfab public database of 3D models of wild ungulates hearts (https://sketchfab.com/KlaraMK/models).

For use in lessons, 3D models were uploaded to a shared online drive, which students were then free to use during their studies. These were 3D models of the hearts of all the species mentioned above. The database of these models was subsequently published in the public database of 3D models on the Sketchfab portal (Figure 8). The database provided students with models of both completely healthy hearts and hearts that had been deformed, for example, by bullet fragments or bone splinters that had damaged the heart muscle as a result, of the individual being shot.

**Figure 9:**
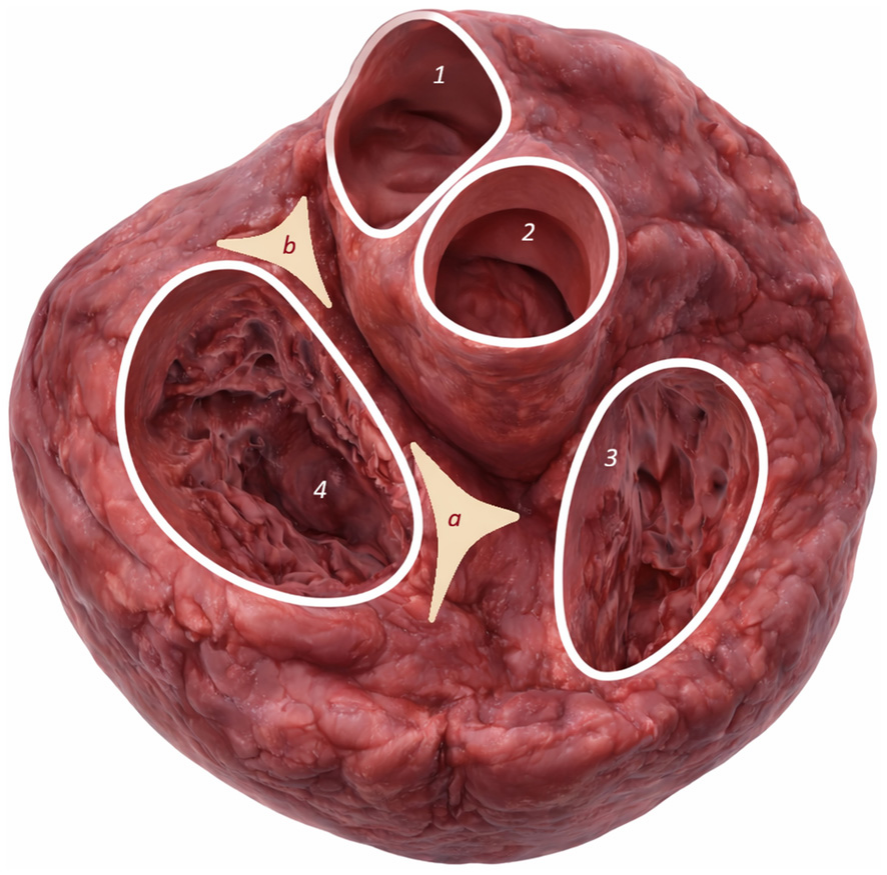
Illustration of the basic anatomical features of the heart for determining the location of the *trigonum fibrosum dextrum et sinistrum* and *Os cordis*. The illustration is based on a 3D model of the heart for teaching purposes. Before CTscanning, the heart was adjusted so that both trigones were exposed. 1–*truncus pulmonalis*, 2–*aorta ascendens*, a – *trigonum fibrosum dextrum*, b – *trigonum fibrosum sinistrum*, 3–*ventriculus dexter*, 4–*ventriculus sinister*.

Students also had access to standard anatomical descriptions of the heart in photographs and a description of the heart created from a 3D model highlighting the *trigonum fibrosum dextrum* and *trigonum fibrosum sinistrum* to provide a simple visual aid for learning the description of the location of the *Os cordis*. (Figure 9).

#### 3.2.1 Student achievement in lessons using 3D models

**Figure 10:**
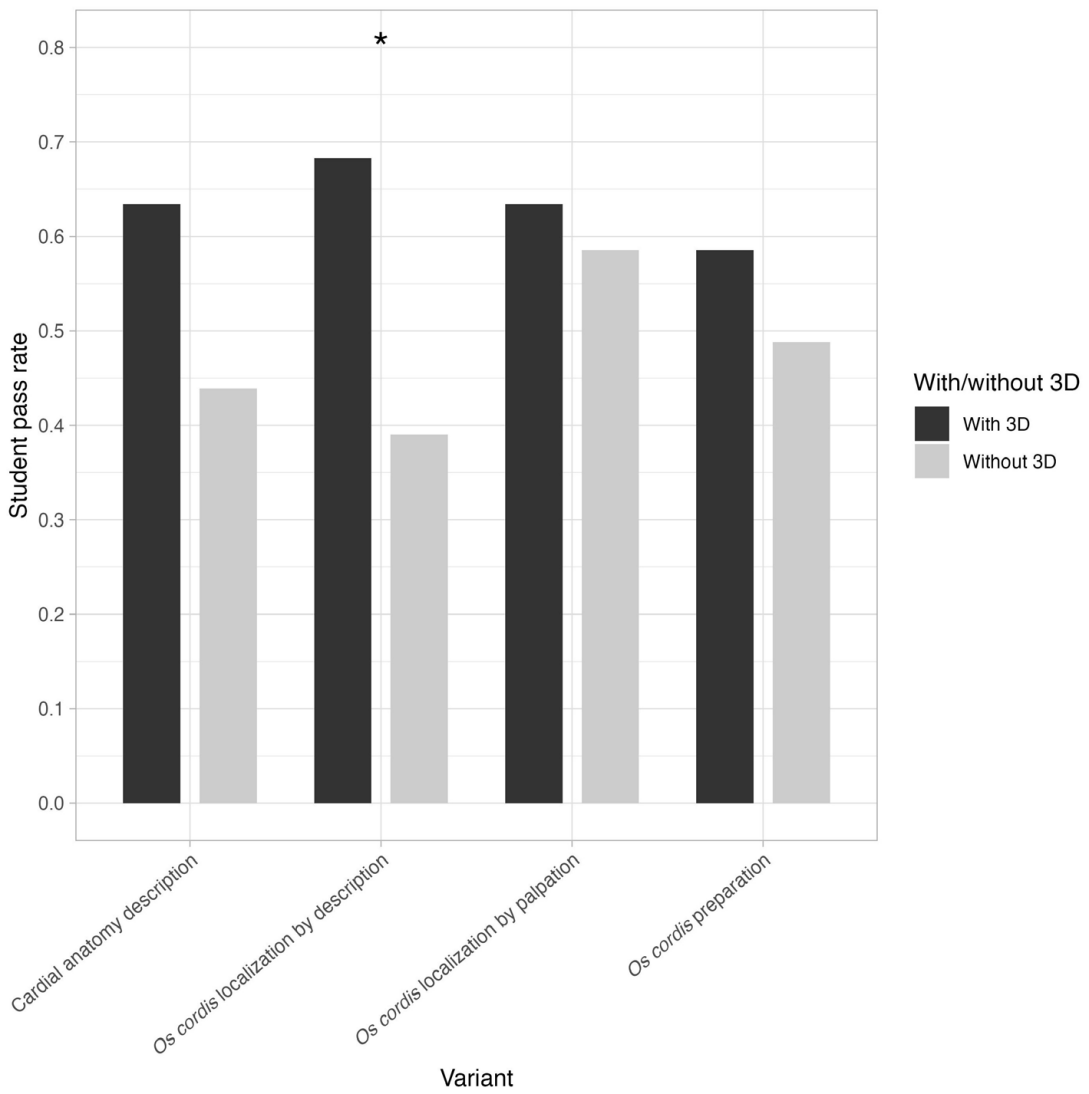
Student examination success rates for selected topics when studying with and without 3D scanner supplementary materials.

For selected tasks, the success rate for adopted topics by the described student group (see Materials and Methods) is presented in Figure 10. The success rate is shown separately for studying with/without 3D supplementary materials. For each variant, the Fischer exact test was performed to test for differences between the success ratio with/without 3D variant. The success rate in describing the anatomy of the heart among students using 3D models reached 63%, compared to only 44% among students who used only 2D graphic materials. The smallest difference between the two groups was recorded in the practical sections of the test, where the 3D group achieved 63% in palpation localization of the *Os cordis*, and the group without 3D models achieved 59%. In the case of dissection itself, the 3D group was 10% more successful, achieving 59%. Significant difference in the success ratio was observed in “*Os cordis* localization by description” (odds ratio = 3.31, 95% CI 1.24–9.25, *P* = 0.014), where the variant “with 3D” showed success ratio of 68.3%, while the variant “without 3D” showed a success ratio of 39.0 %. In other cases, insignificant results were found–Cardial anatomy description: odds ratio = 2.19, 95% CI 0.84 – 5.91, *P* = 0.12; *Os cordis* localization by palpation: odds ratio = 1.22, 95% CI 0.46 – 3.28, *P* = 0.82; *Os cordis* preparation: odds ratio = 1.48, 95% CI 0.57 – 3.89, *P* = 0.51.

## 4 Discussion

### 4.1 Occurrence and localization of *Os cordis* in wild ungulates

The results confirm that the presence of *Os cordis* in ruminant ruminants of Central Europe is predominantly a physiological feature rather than a pathological abnormality. The high incidence of *Os cordis* recorded in red deer, roe deer, fallow deer, and mouflon (86–100%) corresponds to earlier descriptions in domestic and wild ruminants, where *Os cordis* is considered a normal part of the cardiac skeleton (13, 26, 28, 31, 53). In contrast, the complete absence of *Os cordis* in wild boars confirms the fundamental phylogenetic difference between ruminants and non-ruminant mammals, where the cardiac skeleton consists mainly of connective and cartilaginous tissue without mineralization (21, 22, 54). These findings are in line with the fact that the occurrence of *Os cordis* in suids has not yet been confirmed and support the hypothesis that the development of *Os cordis* is related to long-term mechanical stress on atrioventricular structures in ruminants with high body weight and specific hemodynamics (10). In terms of localization, the *Os cordis* was found exclusively in *the trigonum fibrosum dextrum* in all cases, which corresponds to classic anatomical descriptions in cattle, sheep, goats, and deer (23, 24, 26). On the other hand, in some species, such as buffalo (31), the occurrence in the *trigonum fibrosum sinistrum* area is also described, but this was not observed in our research. This uniform localization, even in older individuals, suggests that the species studied do not undergo secondary migration or the formation of ectopic mineralization that could indicate pathological processes, as is the case, for example, in humans or some primates. (35, 36).

### 4.2 The relationship between *Os cordis* morphology and biological parameters of individuals

The density parameters of the *Os cordis*, expressed in Hounsfield units, showed a significant dependence on age, gender, and species in the study. A statistically highly significant increase in density with age (≈ 0.94 HU/month) confirms that the *Os cordis* undergoes a continuous process of mineralization. This finding is fully consistent with histological studies that have demonstrated the transition of hyaline cartilage to bone tissue in older ruminants (10, 13, 55). Similar age-related changes in density have also been described in other ossifying structures, such as the seasonally growing bone tissue of deer antlers (56, 57), which suggests that the *Os cordis* may respond to general regulatory mechanisms of bone metabolism. The higher density of the *Os cordis* in males compared to females may be related to sex-related differences in the hormonal profile, especially the effect of androgens on osteoblastic activity and bone mineralization (58). Significant interspecies differences in the density or size of the *Os cordis*, particularly higher values in European fallow deer and mouflon, may reflect species-specific differences in the hemodynamic load on the heart, heart rate, and relative size of the myocardium. (59–61) state that cardiac output and pressure conditions vary significantly between species of cloven-hoofed animals, which may also influence adaptive changes in the connective tissue and bone skeleton of the heart. In the study, the volume of the *Os cordis* was significantly influenced primarily by the body weight of the individual, with each kilogram of weight resulting in an increase in volume of approximately 0.1 cm3. This relationship supports the hypothesis that the size of the *Os cordis* is closely related to the mechanical demands placed on the cardiac skeleton in larger individuals. However, it is important to note that body weight and overall body build can be significantly influenced by geographical and climatic factors, particularly latitude. In accordance with Bergmann's rule, mammal populations living at higher latitudes generally exhibit larger body sizes than populations from warmer regions (62, 63). In many species, including cervids, there is thus significant geographic variability in body weight and body size (64–66). It is therefore possible that the relationship between an individual's weight and heart volume may also be indirectly influenced by geographic variability among populations. Another aspect affecting body weight could be drived by density-denpendence theory when the body weight is decreasing with the abundance of particular species which is true especially for roe deer (67, 68) however the population increase is described for most of European wild ungulates (5, 6, 69).

### 4.3 The contribution of CT imaging and 3D reconstruction to the differentiation of physiological and pathological changes

The use of a multidetector CT scanner in combination with ethanol fixation has proven to be a very effective method for detecting the *Os cordis* and subtle differences in the density of cardiac structures. A similar approach has also been successfully applied in other studies focusing on the morphology of soft tissues and mineralized structures (23, 29, 38). CT-based volumetry enables standardized comparisons between species and individuals (70) and creates the conditions for long-term monitoring of the health status (71, 72) of wild ungulate populations. In addition, the CT scanner allows clear spatial resolution of the physiological axes of the heart from pathological calcifications, for example, in the area of the valves or myocardium. This ability is notably important for students and less experienced practitioners, who may misinterpret the physiological structure as pathology, as has been described in other studies focused on anatomy teaching (73, 74).

### 4.4 The educational benefits of 3D models in teaching anatomy and pathology

The results of the pedagogical part of the study clearly show that the integration of 3D models into teaching led to improved student performance in all categories monitored, particularly in the anatomical description of the heart and the identification and localization of the *Os cordis*. This effect is consistent with the literature, which repeatedly confirms that spatially interactive models significantly increase understanding of complex anatomical relationships (74–76). The results of this study show that the ability to manipulate a 3D model led to a significant improvement in students' ability to correctly locate the *Os cordis* based on a description, confirming the importance of spatial visualization for understanding this structure. A similar effect has been described for other anatomical areas in veterinary education, where 3D models improve understanding of complex spatial relationships, but not necessarily manual skills themselves (14, 18). The smaller difference between the groups in tasks focused on palpation identification and *Os cordis* dissection can be interpreted as evidence that 3D models do not replace working with biological material, but primarily serve as cognitive support prior to actual manipulation of the real specimen. This conclusion is consistent with general findings from anatomy teaching, according to which digital and physical models work best in combination with traditional dissection methods, not as a substitute for them (16, 76, 77).

### 4.5 Study limitations

Important limitation of the study is the fact that the *Os cordis* was assessed solely on the basis of density values (HU) obtained via CT, without histological verification. Hounsfield units are a reliable indicator of tissue mineral density. Some studies even report that bone density can be directly estimated based on HU without further testing (78). It has also been demonstrated that CT density is sufficiently sensitive to distinguish between different qualities of bone tissue (79), with specific HU thresholds being used in clinical practice as well. These findings suggest that the use of HU as a quantitative parameter is methodologically sound and sufficient for many applications. During scanning, the highest possible accuracy was targeted, which was also verified by control measurements of density parameters on a phantom. However, it should be emphasized that the relationship between HU and the actual microstructure of the tissue is not absolute, and for a detailed description of, for example, the development of the *Os cordis*, it would be desirable to supplement the CT data with a histological analysis of the tissue.

In the educational part of the study, a limiting factor is the design based on a comparison of two different cohorts of students. Therefore, the influence of differences in prior knowledge, motivation, or other didactic factors cannot be ruled out. Furthermore, long-term knowledge retention—which is crucial for assessing the effectiveness of teaching methods—was not evaluated (15, 76). However, the composition of the study group was not influenced by the researcher's selection; given the specificity of the field, similar prior knowledge and group composition can be assumed. The instructional design was developed and implemented directly by the study's author, so we can assert that it was applied exactly as described.

## 5 Conclusion

The high prevalence of *Os cordis* in all ruminants studied and its consistent localization in the *trigonum fibrosum dextrum* confirm that it is a physiological part of the cardiac skeleton and not a pathological change. Conversely, the complete absence of *Os cordis* in wild boar underscores the fundamental differences between ruminants and non-ruminant ungulates, and emphasizes the need for a species-specific approach when interpreting cardiac findings in wild game. Analysis of the morphometric parameters of the *Os cordis* showed a significant dependence on the biological characteristics of individuals.

Methodologically, the combination of ethanol fixation and multidetector CT proved to be a reliable and reproducible approach for studying the fine structures of the cardiac skeleton. CT-based volumetry and 3D reconstruction enabled accurate spatial orientation and clear differentiation of physiological structures from potential pathological calcifications, which is particularly important in teaching and in diagnosis by less experienced evaluators. In addition, digital 3D models allow for standardized comparison of findings across species and studies, and create the conditions for long-term monitoring of the health status of wild ungulate populations. However, without reliable reference data, the presence, morphology, and location of cardiac bones cannot be reliably interpreted either as physiological variations or as signs of a pathological condition, which significantly complicates both scientific interpretation and clinical or postmortem diagnosis. It was therefore important to base teaching materials on relevant data and subsequent findings.

The didactic part of the study showed that the use of interactive 3D models in teaching significantly improves spatial understanding of heart anatomy and students' ability to correctly locate the *Os cordis*. The results suggest that 3D models function primarily as effective cognitive support prior to working with biological material, rather than as a substitute for traditional dissection methods. In fields such as hunting, veterinary hygiene, and wildlife management, digital models are a valuable tool for standardizing teaching and transferring knowledge, even in situations where suitable specimens are not available.

Overall, it can be said that CT-based 3D modeling represents a modern, ethically sustainable, and highly effective supplement to traditional methods of studying anatomy. The data obtained contribute to a better understanding of the functional morphology of the heart in wild mammals and provide a solid foundation for future research into the relationships between cardiac morphology, physiological stress, and the health status of populations.

## Data Availability

The raw data supporting the conclusions of this article will be made available by the authors, without undue reservation.
